# Supplementary material for: The microbiome of the human facial skin is unique compared to that of other hominids
Source: mSystems. 2025 May 28;10(6):e00081-25. doi: 10.1128/msystems.00081-25 (PMC12172416; doi:10.1128/msystems.00081-25)
Supplement: Supplemental material — Supplemental figures and tables. [file msystems.00081-25-s0001.pdf]

Supplementary Section

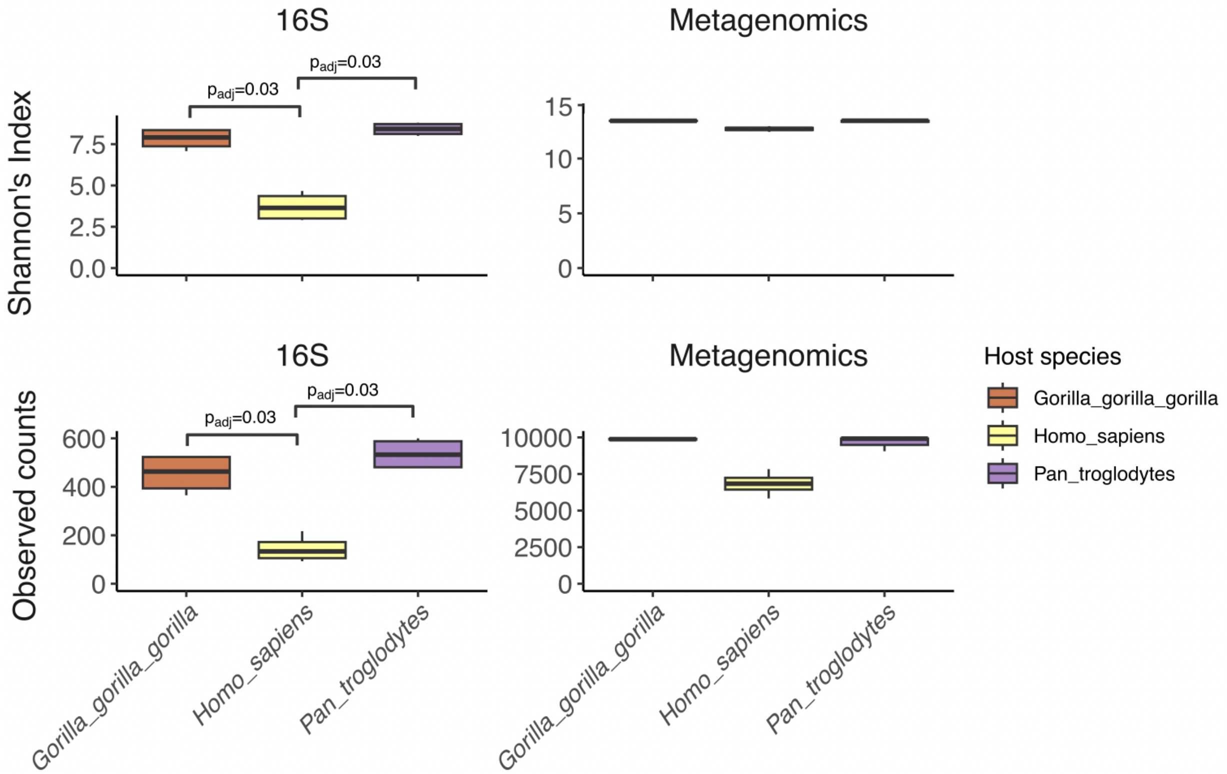

Figure S1. Facial skin microbiome alpha diversity comparisons across hosts. Both Shannon's Index and observed ASV/pathway counts are reported. Data was rarefied prior to analyzing. Bonferroni-corrected P-values denote significant comparisons based on Kruskal-Wallis tests. Sequence data from 16S and shotgun sequencing are reported. A filtering was performed for abundance; therefore count estimates are likely overestimations. Only significant P-values after Bonferroni correction are reported (see Table S2 for further details).

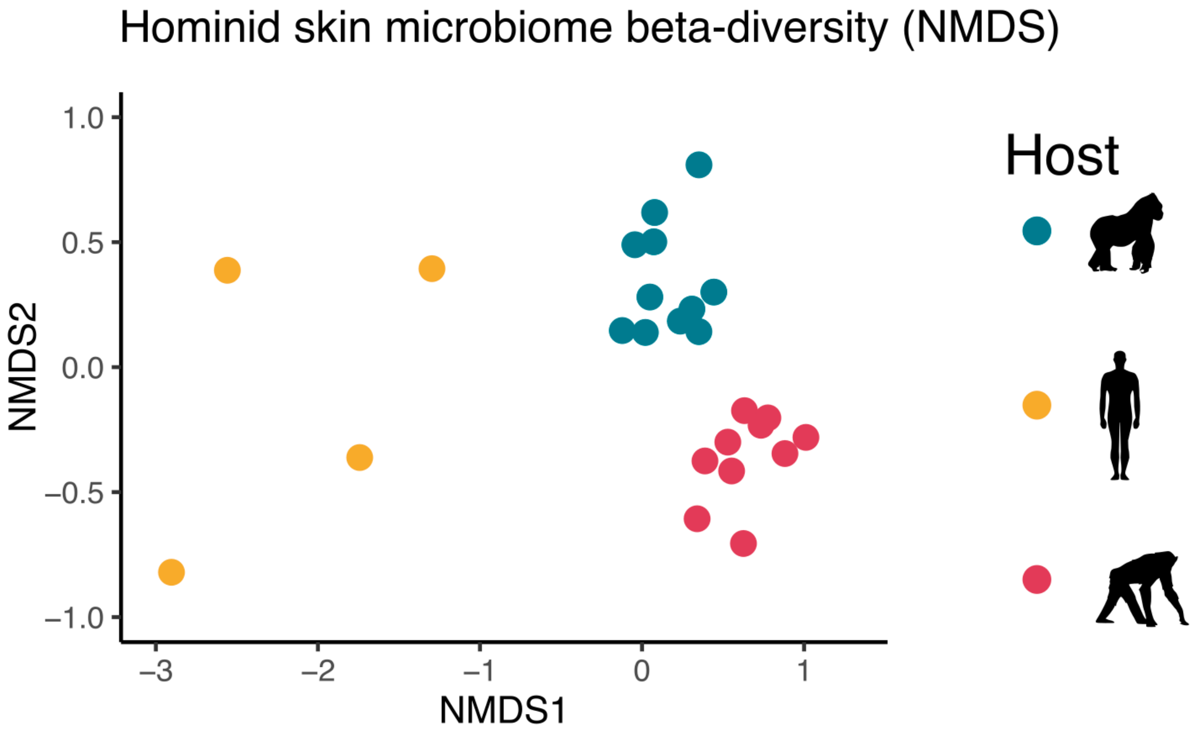

Figure S2. Non-metric multidimensional scaling (NMDS) plots of hominid skin microbiomes (16S) indicates that gorilla and chimpanzee FSMs are more similar to one another than either is to human FSMs. Distances between samples are based on Bray-Curtis distance matrices for both data types. Colors denote host species. Samples are unmerged, thus chimpanzee and gorilla samples consist of cheek, chin, and forehead samples per individual.

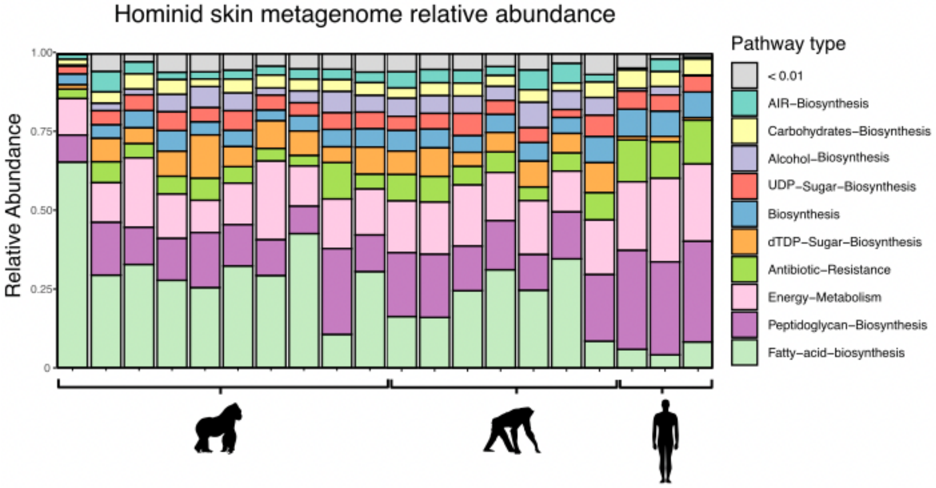

Figure S3. Relative abundance stacked barplot showing pathway composition across hosts. Pathways are collapsed at the type level and pathways under 0.01 relative abundance are binned in the grey bars. Y axis denotes abundance proportions out of 1. NHP samples are unmerged, with forehead, chin, and cheek samples all separated.

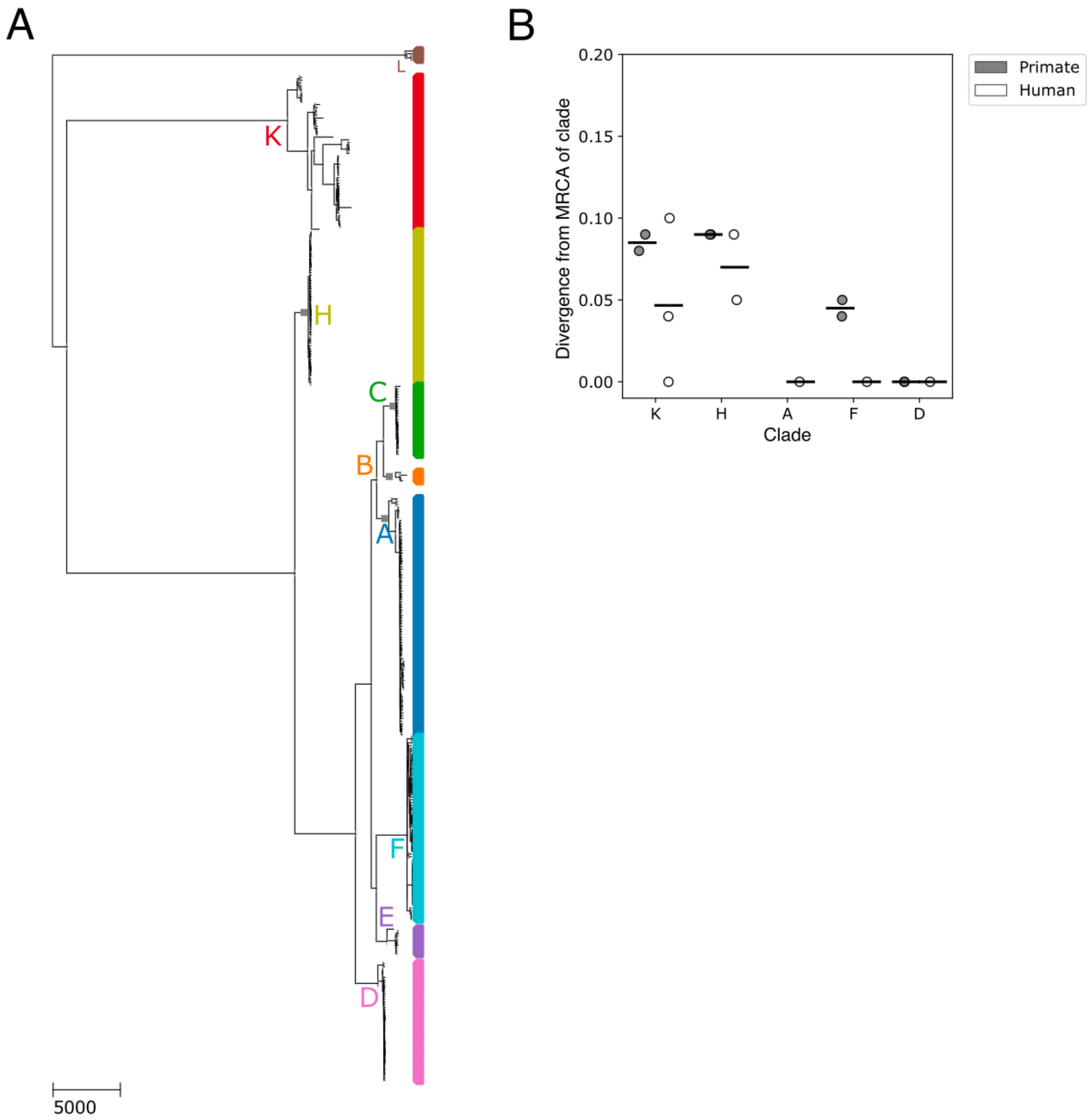

Figure S4. Phylogenomic analysis suggests that *C. acnes* reads from non-human primates have a human origin. A) Maximum likelihood core-genome phylogeny of *C. acnes* was made using 358 public reference genomes isolated from humans. Major phylogenetic clades are labeled as letters according to a conventional typing scheme (Scholz & Kilian et al. 2016) B) Divergence between primate samples and handler samples across clades of *C. acnes* were estimated using PHLAME (https://github.com/quevan/phlame). Only clades with at least one detection are shown. A two-sided Wilcoxon rank-sum test comparing *C. acnes* divergence between NHP and human skin microbiome samples.

Table S1. Effect of host species on skin microbiome composition and function (PERMANOVA).

**16S data**	number of groups	3
	F-test statistic	4.976
	p-value	0.001
	number of permutations	999
	sample size (after merging)	12
**Metagenomic data**	number of groups	3
	F-test statistic	2.487
	p-value	0.001
	number of permutations	999
	sample size (after merging)	12

Table S2. Alpha Diversity Results for 16S and metagenomic (MG) data across host species. NHP samples for each face site are merged per individual to allow more direct comparisons between NHP and human samples.

Group 1	Group 2	H	p-value	p-adj	Data	Metric
Gorilla (n=4)	Homo_sapiens (n=4)	5.333	0.021	0.063	MG	Shannon
Gorilla (n=4)	Pan_troglodytes (n=3)	0	1	1	MG	Shannon
Homo_sapiens (n=4)	Pan_troglodytes (n=3)	3.125	0.077	0.116	MG	Shannon
Gorilla (n=4)	Homo_sapiens (n=4)	5.333	0.0209	0.031	16S	Shannon
Gorilla (n=4)	Pan_troglodytes (n=4)	1.333	0.248	0.248	16S	Shannon
Homo_sapiens (n=4)	Pan_troglodytes (n=4)	5.333	0.021	0.031	16S	Shannon
Gorilla (n=4)	Homo_sapiens (n=4)	5.333	0.021	0.051	MG	Observed
Gorilla (n=4)	Pan_troglodytes (n=3)	0	1	1	MG	Observed
Homo_sapiens (n=4)	Pan_troglodytes (n=3)	4.500	0.034	0.051	MG	Observed
Gorilla (n=4)	Homo_sapiens (n=4)	5.333	0.021	0.032	16S	Observed
Gorilla (n=4)	Pan_troglodytes (n=4)	1.333	0.248	0.248	16S	Observed
Homo_sapiens (n=4)	Pan_troglodytes (n=4)	5.333	0.021	0.031	16S	Observed
All (n=12)		7	0.030	>0.05	MG	Observed
All (n=11)		8	0.018	<0.05	16S	Observed
All (n=11)		8	0.02	<0.05	16S	Shannon
All (n=11)		6.053	0.05	>0.05	MG	Shannon

Table S3. Differential abundance across humans and NHP skin microbiomes (DESeq2 -16S).

Phylum: species	baseMean	log2Fold Change	lfcSE	stat	pvalue	padj
Actinobacteriota: Cutibacterium acnes	19858.237	12.644	0.946	13.370	9.06E-41	7.12E-38
Proteobacteria: Neisseria polysaccharea	112.113	-22.158	2.874	-7.711	1.25E-14	4.92E-12
Actinobacteriota: Lawsonella	1478.979	15.299	2.378	6.433	1.25E-10	3.28E-08
Firmicutes: Anaerococcus nagyae	120.396	11.678	1.994	5.857	4.71E-09	9.26E-07
Bacteroidota: Bacteroidaceae spp	52.104	-7.102	1.357	-5.234	1.66E-07	2.10E-05
Firmicutes: Finegoldia magna	200.216	5.651	1.084	5.212	1.87E-07	2.10E-05
Firmicutes: Staphylococcus haemolyticus	9484.541	10.132	2.104	4.815	1.47E-06	1.45E-04
Proteobacteria:Haemophilus paraphrohaemolyticus	150.080	-6.270	1.313	-4.776	1.78E-06	1.56E-04
Firmicutes: Clostridia spp	58.681	-6.075	1.370	-4.435	9.20E-06	7.23E-04
Firmicutes: Bulleidia massiliensis	83.349	-4.219	0.976	-4.323	1.54E-05	1.01E-03

Table S4. Differential abundance across human and NHP skin metagenomes (DESeq2 - MG)

	baseMean	log2FoldChange	lfcSE	Stat	pvalue	padj
dTDP-Sugar-Biosynthesis	188.368	-2.649	0.308	-8.593	8.49E-18	3.73E-16
Fatty-acid-biosynthesis	905.128	-1.984	0.303	-6.540	6.16E-11	1.36E-09
Peptidoglycan-Biosynthesis	874.155	1.247	0.237	5.263	1.42E-07	2.08E-06
Antibiotic-Biosynthesis	35.169	-2.573	0.502	-5.125	2.97E-07	3.27E-06
Antibiotic-Resistance	360.586	1.287	0.255	5.054	4.32E-07	3.80E-06
Alcohol-Biosynthesis	129.277	-1.748	0.360	-4.853	1.22E-06	8.92E-06
Lipopolysaccharide-Biosynthesis	52.478	-1.765	0.371	-4.754	1.99E-06	1.25E-05
Biosynthesis	305.858	0.968	0.234	4.132	3.59E-05	1.98E-04
Pyrimid-Deoxyribonucleot-Biosyn	24.310	-2.104	0.538	-3.909	9.26E-05	4.53E-04
Ascorbate-Biosynthesis	130.367	1.049	0.296	3.541	3.98E-04	1.64E-03
Choline-Degradation	11.940	-3.614	1.027	-3.518	4.36E-04	1.64E-03
Sucrose-Biosynthesis	129.435	1.051	0.299	3.511	4.47E-04	1.64E-03
Energy-Metabolism	860.909	0.840	0.257	3.266	1.09E-03	3.69E-03
Aromatic-compounds-degradation	18.962	-2.123	0.666	-3.188	1.43E-03	4.50E-03

*negative log2fold changes denote pathways that are found in higher proportions in NHP skin microbiomes whereas positive changes are pathways that are found in higher proportions in human skin microbiomes.

Table S5. Kruskal-Wallis analysis on fatty-acid biosynthesis pathway relative abundance across humans and NHP skin metagenomes

Pathway	Path ID	Group 1	Group 2	n1	n2	statistic	p	padj
Palm Biosynthesis 1	PWY.5994	Gorilla	human	12	4	-3.677	0.000	0.001
Palm Biosynthesis 1	PWY.5994	Gorilla	Pan_troglodytes	12	8	-2.326	0.020	0.040
Palm Biosynthesis 1	PWY.5994	human	Pan_troglodytes	4	8	1.734	0.083	0.083
Mycolate Biosynthesis	PWYG.321	Gorilla	human	12	4	-3.348	0.001	0.002
Mycolate Biosynthesis	PWYG.321	Gorilla	Pan_troglodytes	12	8	-1.756	0.079	0.129
Mycolate Biosynthesis	PWYG.321	human	Pan_troglodytes	4	8	1.848	0.065	0.129
Palm Biosynthesis 2	PWY.5971	Gorilla	human	12	4	-3.143	0.002	0.005
Palm Biosynthesis 2	PWY.5971	Gorilla	Pan_troglodytes	12	8	-1.110	0.267	0.267
Palm Biosynthesis 2	PWY.5971	human	Pan_troglodytes	4	8	2.136	0.033	0.065

Table S6. Wilcoxon test results comparing pairwise distance values across comparisons of chimpanzee, gorilla, and human skin microbiomes.

Comparison 1	Comparison 2	p-value	W-statistic	Data type
Chimp:human	Gorilla:chimp	P=4.81e-04	W=136	16S
Chimp:human	Gor:human	P=0.598	W=142.5	16S
Gorilla:chimp	Gor:human	P=1.53e-06	W=0	16S
Chimp:human	Gorilla:chimp	P=5.56e-03	W=120.5	Pathway
Chimp:human	Gor:human	P=0.114	W=61.5	Pathway
Gorilla:chimp	Gor:human	P=2.65e-06	W=5	Pathway

Table S7. Sequences of the 16S rRNA gene primers used. Standard primers were used for both the V1-V3 and the V3-V4 regions of the 16S rRNA gene.

V1-V3 27F-plex	5'TCGTCGGCAGCGTCAGATGTGTATAAGAGACAGAGAGTTTGATCMTGGCTCAG
V1-V3 534R-plex	5'GTCTCGTGGGCTCGGAGATGTGTATAAGAGACAGATTACCGCGGCTGCTGG

Table S8: BLAST results of the top hit for metagenome-assembled contigs that had a best hit to a *Cutibacterium* spp.

Contig Name	Taxonomy	PercId	Alignment Length	Evalue
NODE_62_length_20193_cov_7.390506	Cutibacterium modestum	100	78	2.72E-28
NODE_62_length_20193_cov_7.390506	Cutibacterium acnes	100	78	2.72E-28
NODE_10224_length_523_cov_2.038462	Cutibacterium avidum	96.839	348	4.23E-161
NODE_423_length_1920_cov_2.136729	Cutibacterium acnes	81.916	1493	0
NODE_3329_length_504_cov_1.207127	Cutibacterium granulosum	91.778	523	0

Table S9. Identified sequences (Sanger) from *Cutibacterium* culturing attempts from primate facial skin swabs.

bp_score	identity	quality	startpos	stoppos	ecolipos	bps	LCA taxonomic assignment (silva)
98	98.7842	97	1050	32660	31	999	Staphylococcus; unassigned
97	98.509	96	1055	32544	33	1000	Staphylococcus; unassigned
98	99.8951	98	1040	32512	26	1000	Enterococcus; unassigned
100	98.4488	98	1055	32663	33	1000	Staphylococcus; unassigned
97	95.5524	96	1087	22114	47	713	Staphylococcus; unassigned
100	99.4893	97	1045	32549	29	999	Staphylococcus; unassigned
100	99.3909	98	1055	32549	33	1000	Staphylococcus; unassigned
104	96.5429	93	1059	26856	35	840	Staphylococcus; unassigned
99	98.617	96	1059	32538	35	1000	Staphylococcus; unassigned
86	96.1938	92	1055	16286	33	599	Staphylococcus; unassigned
100	98.881	97	1050	32525	31	1000	Staphylococcus; unassigned
99	99.3909	98	1059	32544	35	1000	Staphylococcus; unassigned
97	99.2915	98	1059	32663	35	1000	Staphylococcus; unassigned
101	99.4647	98	1052	32538	32	1000	Staphylococcus; unassigned
98	99.1498	98	1055	32663	33	1000	Staphylococcus; unassigned
96	98.2997	96	1057	32551	34	1000	Staphylococcus; unassigned
94	95.7747	91	1050	7696	31	348	Staphylococcus; unassigned
96	95.8724	92	1059	15662	35	554	Staphylococcus; unassigned
99	99.0405	97	1055	32544	33	1000	Staphylococcus; unassigned
99	97.3795	96	1041	32551	26	1000	Staphylococcus; unassigned
97	98.7275	97	1057	32664	34	1000	Staphylococcus; unassigned
98	99.361	98	1068	32551	39	1000	Staphylococcus; unassigned
99	99.1886	97	1057	32549	34	1000	Staphylococcus; unassigned
98	99.2561	98	1057	32663	34	1000	Staphylococcus; unassigned
99	98.9848	97	1055	32549	33	1000	Staphylococcus; unassigned
87	89.749	84	1045	32663	29	991	Staphylococcus; unassigned
